# Dectin-1 and DC-SIGN Polymorphisms Associated with Invasive Pulmonary Aspergillosis Infection

**DOI:** 10.1371/journal.pone.0032273

**Published:** 2012-02-27

**Authors:** Juan Sainz, Carmen Belén Lupiáñez, Juana Segura-Catena, Lourdes Vazquez, Rafael Ríos, Salvador Oyonarte, Kari Hemminki, Asta Försti, Manuel Jurado

**Affiliations:** 1 Genomic Oncology Area, Genyo (Pfizer-University of Granada-Andalusian Government Centre for Genomics and Oncological Research), Granada, Spain; 2 Molecular Genetic Epidemiology Department, German Cancer Research Center, Heidelberg, Germany; 3 Haematology Department, University Hospital Virgen de las Nieves, Granada, Spain; 4 Hematology Department, University Hospital of Salamanca, Salamanca, Spain; 5 Blood Transfusion Regional Centre and Sectorial Tissue Bank, Granada, Spain; 6 Center for Primary Health Care Research, Clinical Research Center, Malmö, Sweden; University of Kentucky, United States of America

## Abstract

The recognition of pathogen-derived structures by C-type lectins and the chemotactic activity mediated by the CCL2/CCR2 axis are critical steps in determining the host immune response to fungi. The present study was designed to investigate whether the presence of single nucleotide polymorphisms (SNPs) within *DC-SIGN*, *Dectin-1*, *Dectin-2*, *CCL2* and *CCR2* genes influence the risk of developing Invasive Pulmonary Aspergillosis (IPA). Twenty-seven SNPs were selected using a hybrid functional/tagging approach and genotyped in 182 haematological patients, fifty-seven of them diagnosed with proven or probable IPA according to the 2008 EORTC/MSG criteria. Association analysis revealed that carriers of the *Dectin-1*_rs3901533 T/T_ and *Dectin-1*_rs7309123 G/G_ genotypes and *DC-SIGN*_rs4804800 G_, *DC-SIGN*_rs11465384 T_, *DC-SIGN*_7248637 A_ and *DC-SIGN*_7252229 C_ alleles had a significantly increased risk of IPA infection (OR = 5.59 95%CI 1.37–22.77; OR = 4.91 95%CI 1.52–15.89; OR = 2.75 95%CI 1.27–5.95; OR = 2.70 95%CI 1.24–5.90; OR = 2.39 95%CI 1.09–5.22 and OR = 2.05 95%CI 1.00–4.22, respectively). There was also a significantly increased frequency of galactomannan positivity among patients carrying the *Dectin-1*_rs3901533_T_ allele and *Dectin-1*_rs7309123_G/G_ genotype. In addition, healthy individuals with this latter genotype showed a significantly decreased level of Dectin-1 mRNA expression compared to C-allele carriers, suggesting a role of the *Dectin-1*_rs7309123_ polymorphism in determining the levels of Dectin-1 and, consequently, the level of susceptibility to IPA infection. SNP-SNP interaction (epistasis) analysis revealed significant interactions models including SNPs in *Dectin-1*, *Dectin-2, CCL2 and CCR2* genes, with synergistic genetic effects. Although these results need to be further validated in larger cohorts, they suggest that *Dectin-1, DC-SIGN, Dectin-2, CCL2 and CCR2* genetic variants influence the risk of IPA infection and might be useful in developing a risk-adapted prophylaxis.

## Introduction

Invasive Pulmonary Aspergillosis (IPA) is a life-threatening infection caused mainly by *Aspergillus fumigatus*, an opportunistic fungal pathogen that frequently colonizes respiratory tracts and rapidly spreads to blood vessels and tissues [1]. The incidence of IPA infection has increased in the last years due to the use of immunosuppressive and immunomodulatory drugs and is still causing significant morbidity and mortality worldwide, especially in immunosuppressed and hematopoietic stem cell transplantation (HSCT) patients [2].

Early recognition of *A. fumigatus* by myeloid leukocytes is a crucial for down-stream immune response and conidia clearance and, therefore, in the development and progression of IPA infection [3]. Myeloid leukocytes are activated by pattern-recognition receptors (PRRs), which directly recognize fungal cell wall structures and pathogen-associated molecular patterns (PAMPs) [4]. Among the PRRs expressed in neutrophils, pulmonary macrophages and dendritic cells, C-type lectin family members such as *DC-SIGN* (Dendritic Cell-Specific Intercellular adhesion molecule-3-Grabbing Non-integrin), *Dectin-1* (Dendritic cell-associated C-type lectin-1) and *Dectin-2* (Dendritic cell-associated C-type lectin-2) seem to be important in the effectiveness of innate immune response against *A. fumigatus* [5], [6]. Recent studies have reported that the C-type lectins mediate *A. Fumigatus* recognition, capture and internalization [7] and that their binding to fungal cell wall carbohydrates is highly specific and selective [8]. *DC-SIGN* and *Dectin-1* recognize β-(1-3)-glucans and galactomannans (GM) in the cell wall of *A. fumigatus* [7], [9] while *Dectin-2* interacts with α-mannans [10]. However, numerous studies have shown that C-type lectins are not only involved in the recognition of fungal pathogens but also in the induction of anti-fungal Th1 and Th17 immune responses [5], [11].

Although the mechanisms underlying *DC-SIGN*-mediated immune response are still highly speculative, it has been suggested that *DC-SIGN* promotes dendritic cell (DC) migration and T-cell activation through the ICAM-3 binding [12], [13] and modulates *TLR* signalling by targeting *Raf-1*, which regulates NFκB p65 activation and, consequently the production of pro-inflammatory cytokines [14]. Likewise, *Dectin-1* and *Dectin-2* induce Syk-dependent canonical and noncanonical *NFκB* pathways [15], [16] promoting the production of some pro-inflammatory cytokines (*IL1α*, *IL1β*, *IL12* and *IL8*), chemokines (*CCL2/MCP-1*, *CCL3/MIP-1*, *CXCL2/MIP-2* and *CXCL10*) [17] and the IL17-mediated neutrophil recruitment [18]. It has also been described that *Dectin-1* and *Dectin-2* are able to work in collaboration with *TLRs* (mainly *TLR2*, *TLR4* and *TLR6*) modulating immune responses through the production of *IL6*, *IL12p70* and *TNF* [3], [16]. These findings support the hypothesis that C-type lectins, cytokines or chemokines are important mediators during infection with *A. fumigatus*.

Several polymorphisms in human PRRs [19]–[24] as well as in some cytokines [25]–[27], chemokines [28] and their receptors [29], [30] have hitherto been associated with an increased risk of invasive aspergillosis in susceptible hosts. Based on these observations, the objective of the present study was to investigate the role of tagging and potentially functional single-nucleotide polymorphisms (SNPs) located within the *DC-SIGN*, *Dectin-1*, *Dectin-2*, *MCP-1/CCL2* and *CCR2* genes on IPA susceptibility.

## Materials and Methods

### Study population and clinical diagnosis of IPA infection

All participants enrolled were Caucasian and recruited in the University Hospital Virgen de las Nieves (Granada, Spain) or in the University Hospital of Salamanca (Salamanca, Spain). All determinations and genetic analyses in hematological patients were performed with fully informed written consent, and anonymity of the data was guaranteed. The study protocol was approved by the Ethical Review Committee of Virgen de las Nieves University Hospital, Granada, Spain. The population included 182 hematological patients recruited between January 2004 and January 2011. All hematological patients in this study received a prolonged chemotherapy treatment or underwent HSCT and were therefore considered susceptible to develop IPA infection. Demographic information and clinical data were obtained by detailed review of hospital records. Data were gathered on: site of infection; host factor criteria (severe neutropenia for >10 days, persistent fever for >96 h refractory to appropriate broad-spectrum antibacterial treatment, signs and symptoms indicating graft-versus-host disease [GVHD], corticoid therapy [>0.3 mg/kg per day], and invasive fungal infection during a previous episode of neutropenia), microbiological criteria (positive result for *Aspergillus* antigen in **≥**2 consecutive blood samples when considering an index of 0.5 or in only 1 sample when the index was higher than 0.8), and clinical criteria of lower respiratory tract infection [major criteria: any of the following new infiltrates on computed tomography (CT) imaging: halo sign, air-crescent sign, or cavity within area of consolidation; minor criteria: cough, thoracic pain, hemoptysis, pathologic pulmonary sound, and radiological evidence suggestive of invasive infection]. Laboratory data were also recorded. Proven and probable IPA was diagnosed based on the updated criteria (2008) reported by the European Organization for Research and Treatment of Cancer/Invasive Fungal Infections Cooperative Group (EORTC/IFICG) [31].

### Quantification of serum galactomannan antigen

Serum GM detection has been shown to be a useful test for the early diagnosis and follow-up of IPA and is now included in IPA diagnosis criteria [31]. In the present study, serum GM antigen was determined twice weekly during the hospital stay and at each outpatient visit until the end of their immunosuppressant or chemotherapeutic treatment. Serum GM concentrations were determined by Platelia Aspergillus ELISA kit (Bio-Rad, Marnes-la-Coquette, France) according to the manufacturer**'**s instructions. This commercial kit has proven to offer good sensitivity to detect GM [32], and GM concentration was found to correlate with the fungal tissue burden [33], [34]. A test sample was classified as positive when the optical density ratio was ≥0.5 in two consecutive positive samples or >0.8 in a unique serum sample. A careful review of concomitant treatments (piperacillin-tazobactam or amoxicillin-clavulonic acid) in each patient was necessary to detect possible false-positive GM determinations. Likewise, tests were performed on the same day to avoid sample contamination and to ensure accuracy of results.

### SNP selection and genotyping

Twenty-seven tagging/functional SNPs within *DC-SIGN*, *Dectin-1*, *Dectin-2*, *CCL2* and *CCR2* were selected to genotype the entire panel of individuals (Table 1). The aim of the SNP tagging was to identify a set of SNPs that efficiently tags all the known SNPs while the functional approach was used to determine the net impact of potentially functional variants within *DC-SIGN, Dectin-1, Dectin-2, CCL2 and CCR2* genes on IPA risk. Tagging SNPs were selected using Haploview Tagger program (http://www.broad.mit.edu/mpg/haploview/; http://www.broad.mit.edu/mpg/tagger/) and a pairwise tagging with a minimum r2 of 0.8. In this selection we forced the inclusion of the *DC-SIGN*_rs4804803_, *CCL2*_rs1024610_ and *CCL2*_rs1024611_ polymorphisms as their functionality has been suggested [35]–[37]. Genomic DNA was extracted from peripheral blood mononuclear cells (PBMCs) Qiagen Mini Kit (Qiagen, Valencia, CA, USA). Genotyping of *DC-SIGN*, *Dectin-1*, *Dectin-2*, *CCL2* and *CCR2* polymorphisms was carried out using KASPar assays (KBiosciences, Hoddesdon, Hertfordshire, UK) in a 384-well plate format (Applied Biosystems, Foster City, CA, USA) where hematological patient samples were randomly distributed. KASPar reactions were performed using KASPar assay mix (containing probes) and KASPar kit containing 2× Reaction Mix and MgCl2 (50 mM). PCR conditions were: denaturation at 94°C for 15 min, 10 cycles of denaturation at 94°C for 10 sec, annealing at 57°C for 5 sec and elongation at 72°C for 10 sec and 20 cycles of denaturation at 94°C for 10 sec, annealing at 57°C for 20 sec and elongation at 72°C for 40 sec. Recycling conditions were 94°C for 10 sec, annealing and elongation at 60°C for 60 sec. PCR products were analyzed with the ABI Prism 7900HT detection system using the SDS 2.4 software (Applied Biosystems). For internal quality control, about 5% of samples were randomly selected and included as duplicate. Concordance between the original and the duplicate samples for the 27 SNPs analyzed was ≥99.5%. Call rates for all SNPs were ≥97.8% with the exception of the *Dectin-1*_rs11053599_ SNP with a call rate of 94.5%.

**Table 1:** Selected SNPs within DC-SIGN, Dectin-1, Dectin-2, CCL2 and CCR2 genes.

Gene and SNP position	dbSNP rs#	Location	Aa change	Nucleotide substitution	Hypothetical function and/or reported associations	Reference
DC-SIGN_c.−139	rs2287886	Promoter	-	A/G	Affects transcriptional activity and DC-SIGN mRNA expression level; associated with protection against IPA infection; associated with several infection and immune-related diseases such as HIV-1, Dengue, TB, parenteral infection, SARS, RA.	Sainz et al. 2010; Chang *et al.* 2010; Koizumi *et al.* 2007; Mezger et al. 2008;Kashima *et al.* 2009; Martin *et al.* 2004;Gómez *et al.* 2006
DC-SIGN_c.−336	rs4804803	Promoter	-	A/G	Affects transcriptional activity and DC-SIGN mRNA expression; associated with risk of parenteral infection, HIV-1, HCV, dengue and tuberculosis.	Sakuntabhai *et al.* 2005; Martin *et al.* 2004Barreiro *et al.* 2006; Ryan *et al*. 2010
DC-SIGN_c.2797	rs4804800	3′-UTR	-	A/G	3′-UTR affecting RNA expression	-
DC-SIGN_c.342+2863	rs8112310	5′ near gene	-	A/T	Potential activity affecting DC-SIGN expression	-
DC-SIGN_IVS6 −326	rs10410342	Intron	-	C/G	Unknown	-
DC-SIGN_ c.749−28	rs11465384	3′-UTR	-	C/T	3′-UTR affecting RNA expression	-
DC-SIGN_ c.1974	rs11465413	3′-UTR	-	A/T	3′-UTR affecting RNA expression	-
DC-SIGN_IVS2+11	rs7252229	Intron	-	G/C	Unknown	-
DC-SIGN_c.898	rs7248637	3′-UTR	-	A/G	3′-UTR affecting RNA expression	-
DC-SIGN_c.2629	rs11465421	3′-UTR	-	A/C	3′-UTR affecting RNA expression	-
Dectin-1 (CLEC7A)_c.714	rs16910526	Coding exon	Y238X	A/C	Defective expression and lack of b-glucan recognition byPhagocytes; associated with increased *Aspergillus* and *Candida* colonization in hematopoietic transplant recipients	Ferwerda *et al.* 2009Cunha *et al.* 2010; Platinga *et al.* 2009
Dectin-1 (CLEC7A)_c.375−1148	rs11053599	Intron	-	A/C	Unknown	-
Dectin-1 (CLEC7A)_c.375−1404	rs7309123	Intron	-	C/G	Unknown	-
Dectin-1 (CLEC7A)_c.255+813	rs3901533	Intron	-	G/T	Unknown	-
Dectin-1 (CLEC7A)_c.104−520	rs4763446	Intron	-	C/T	Unknown	-
Dectin-1 (CLEC7A)_c.104−811	rs16910631	Intron	-	C/T	Unknown	-
Dectin-1 (CLEC7A)_c.103+732	rs7311598	Intron	-	A/G	Unknown	-
Dectin-2 (CLEC6A)_c.369+338	rs7134303	Intron	-	A/G	Unknown	-
Dectin-2 (CLEC6A)_c.122−425	rs4264222	Intron	-	C/T	Unknown	-
Dectin-2 (CLEC6A)_c.32−699	rs4459385	Intron	-	C/T	Unknown	-
CCL2 (MCP-1)_c.903	rs4586	Coding exon	C35C	C/T	Associated with an increased risk of TB	Thye *et al.* 2009
CCL2 (MCP-1)_c.−2136	rs1024610	Promoter	-	A/T	Unknown	-
CCL2 (MCP-1)_c.−2518	rs1024611	Promoter	-	C/T	Correlate with MCP-1 mRNA expression; associated with increased risk to TB, HCV and HBV infections	Ganachari et al. 2010; Park et al. 2006;Flores-Villanueva *et al.* 2005; Mühlbauer et al. 2003
CCL2 (MCP-1)_c.1543	rs13900	3′-UTR	-	C/T	Unknown	-
CCR2 _c.−1221	rs3918358	Promoter	-	A/C	Unknown	-
CCR2 _c.667	rs743660	3′-UTR	-	A/G	Associated with a decreased risk of Asthma	Kim *et al.* 2007
CCR2_Ex2+241	rs1799864	Coding exon	V64I	A/G	Associated with slower progression to HIV	Smith *et al.* 1997

Abbreviations: UTR, untranslated region; TB, Tuberculosis; HCV, Hepatitis C virus; HBV, Hepatitis B virus; HIV-1, Human immunodeficiency virus-1; SARS, acute severe respiratory syndrome; RA, Rheumatoid arthritis.

### Statistical analysis

The Hardy-Weinberg Equilibrium (HWE) tests were performed in the control group (non-IPA patients) by a standard observed-expected chi-square (χ^2^) test at each polymorphic site (http://ihg2.helmholtz-muenchen.de/cgi-bin/hw/hwa1.pl). Unconditional logistic regression was used to assess the main effects of the genetic polymorphisms on IPA risk using co-dominant, dominant and recessive inheritance models. For each SNP, the more common allele in the control group was assigned as the reference category. All analyses were adjusted for age, gender, hematological malignancy and established risk factors for IPA infection (HSCT, neutropenia, GVHD and corticoid therapy use) and were conducted using the statistical software SSPS (version 14.0, SPSS Inc., Chicago, USA). All tests were considered to be statistically significant with a p value of <0.05.

Adjustment for multiple testing was carried out following a conservative threshold for statistical significance, based on a revised version of the Bonferroni method: we calculated for each gene the “number of effective independent variables” (M_eff_) by use of the SNP Spectral Decomposition approach (http://gump.qimr.edu.au/general/daleN/SNPSpDsuperlite/) [38]. We obtained a study-wise M_eff_ value by adding up the gene M_eff_'s.

SNPtool (http://www.dkfz.de/de/molgen_epidemiology/tools/SNPtool.html) [39] and the Haploview v4.2 software were used for LD blocks reconstruction and haplotype association statistics. Block structures were determined according to the method of Gabriel et al. [40].

### Functional prediction of associated SNPs

We used the Web-based tool FastSNP [41] available at http://fastsnp.ibms.sinica.edu.tw for predicting the functional significance of the SNPs associated with IPA infection. FastSNP utilizes information from another online program PolyPhen (http://www.bork.embl-heidelberg.de/PolyPhen/) and from four different web resources (TFSearch, ESEfinder, Rescue-ESE and FAS-ESS) to determine whether SNPs are located at exonic splicing regulatory sites, cause a non-conservative amino acid change or whether they alter the transcription factor-binding site of a gene (for instance, acting as intronic enhancer). The score was given by this tool on the basis of levels of risk with a ranking of 0 (no effect), 1 (very low), 2 (low), 3 (medium), 4 (high), or 5 (very high).

### RNA extraction, reverse transcription, and qPCR

Whole blood samples from 21 healthy donors were collected into PAXGENE RNA tubes and stored at −80°C until use. Total RNAs were extracted using a PAXGENE Blood RNA Isolation Kit (PreAnalytiX) and reverse transcribed to cDNA using QuantiTect Reverse Transcription Kit (catalog number: 205311; Qiagen). Real-time quantitative PCR was carried out using an ABI PRISM® 7500 HT Sequence Detection System (Applied Biosystems) according to manufacturer's instructions. Briefly, 2 µl of the cDNA were loaded in a PCR reaction containing 12.5 µl of 2× QuantiTect SYBR Green PCR Master Mix with an appropriate concentration of MgCl_2_, 2.5 µl of primers (Hs_CLEC7A_1_SG QuantiTect Primer Assay, catalog number: QT00024059; Geneglobe, Qiagen) and 8 µl of RNase-free water. PCR cycling conditions were as follows: 95°C for 15 minutes, followed by 40 cycles of denaturation at 95°C for 15 seconds combined with annealing at 60°C for 30 seconds and extension at 72°C for 30 seconds. All samples were run in duplicate. Relative quantification of Dectin-1 mRNA expression was calculated with the 2^−ΔΔCt^ method. We obtained the fold changes in gene expression normalized to an internal control gene (GAPDH, Hs_GAPDH_2_SG QuantiTect Primer Assay, Catalog number: QT01192646; Qiagen) and relative to one calibrator (FirstChoice®Human Brain Reference RNA, catalog number: 6050; Applied Biosystems).

### SNP-SNP interaction analysis

We also analyzed high-order interactions between SNPs using the multifactor dimensionality reduction (MDR) constructive induction algorithm. A detailed explanation on the MDR method has been described elsewhere [42], [43]. SNPs in LD (cut-off of r^2^∼0.8) were excluded from the MDR analysis. Cross-validation and permutation testing were used to identify the best models. All possible two-, three- and four-way SNP interactions were tested using 100-fold cross-validation and the exhaustive search. The model with the highest testing balanced accuracy (TA) and cross validation consistency (CVC) was selected as “best model”. Statistical significance was evaluated using a 1.000-fold permutation test to compare observed testing balanced accuracies with those expected under the null hypothesis of no association (using the MDR permutation testing module 0.4.9 alpha). MDR results were considered statistically significant at the 0.05 level. Finally, interactions were visualized by constructing an interaction dendrogram according to the method described by Moore et al [43]. MDR software and MDR permutation testing module are open-source and freely available from http://www.epistasis.org.

## Results

Population characteristics are described in Table 2. Compared to non-IPA, IPA cases were more likely to have cough and pathologic pulmonary sound (p<0.001 and 0.011, respectively) and presented more often pathological chest radiographies and CT scans (p<0.001). Established risk factors for IPA infection such as HSCT, neutropenia, GVHD and corticoid therapy use were homogenously distributed between IPA and non-IPA patient groups.

**Table 2:** Demographic and clinical data of IPA and non-IPA patients.

	Patients with IPA (n = 57)	Patients without IPA (n = 125)	‡ *P* values
Demographic variables			
Age (range)	48.98 (16-76)	50.95 (16-78)	NS
Sex ratio (male/female)	35/22	70/55	NS
Hematological disease (%)			
AML	27 (47.37)	41 (32.80)	
ALL	9 (15.79)	12 (9.60)	
MM	4 (7.02)	20 (16.00)	
CML	0 (0.00)	2 (1.60)	
HL	2 (3.51)	15 (12.00)	
NHL	8 (14.03)	23 (18.40)	
AA	3 (5.26)	2 (1.60)	
CLL	2 (3.51)	6 (4.80)	
MDS	2 (3.51)	4 (3.20)	
EORTC/MSG 2008 classification			
Proven IPA	13 (22.80)	-	
Probable IPA	44 (77.20)	-	
Risk factors			
HSCT	33 (57.90)	53 (42.4)	NS
Severe neutropenia†	45 (78.95)	105 (84.00)	NS
Corticoid therapy	14 (24.56)	40 (32.00)	NS
GVHD	8 (14.04)	11 (8.80)	NS

Abbreviations: NS, non-significant. HSCT: Hematopoietic stem cell transplantation, AML: acute myeloid leukemia, ALL: acute lymphoid leukemia, MM: Multiple Myeloma, CML: chronic myeloid leukemia, HL: Hodgekin's lymphoma, NHL: non-Hodgekin's lymphoma, AA: Aplastic anemia, CLL: chronic lymphocytic leukemia, MDS: myelodysplastic syndrome.

† : Severe neutropenia was defined as absolute neutrophil count <500 cells/mm^3^ for a period of more than 12 days. GVHD: graft versus host disease;

‡ : p-values were calculated by student-t test for continous- and Fishers exact test for binary data.

Fifty-seven patients were diagnosed with proven or probable IPA infection according to the revised EORTC/MSG criteria (2008). The association of risk of IPA infection with the individual 27 SNPs in the C-type lectin and chemokine genes is shown in the Table S1. All analyzed polymorphisms fulfilled Hardy-Weinberg expectations for the control group (non-IPA patients).

Several polymorphisms were found to be associated with IPA infection (Table 3). The *Dectin-1*_rs3901533_, *Dectin-1*_rs7309123_ and *DC-SIGN*_rs4804800_ polymorphisms showed the strongest association with risk of IPA infection (OR_T/T_ = 5.59, 95%CI1.37–22.77; OR_G/G_ = 4.91, 95%CI1.52–15.89 and OR_A/G+G/G_ = 2.75, 95%CI 1.27–5.95, respectively). Additionally, carriers of the *DC-SIGN*_rs11465384_T_, *DC-SIGN*_7248637_A_ and *DC-SIGN*_7252229_C_ alleles had a significantly increased risk of IPA infection (OR_C/T+T/T_ = 2.70, 95%CI 1.24–5.90; OR_G/A+A/A_ = 2.39, 95%CI 1.09–5.22; OR_G/C+C/C_ = 2.05, 95%CI 1.00–4.22, respectively) whereas patients carrying the *DC-SIGN*_rs2287886_A_ allele showed a tendency to be associated with a decreased risk of IPA infection (per-allele OR = 0.60, 95%CI 0.34–1.05) (Table 3 and Table S1). After correction for multiple testing using SNPSpD (number of independent marker loci, 21; p = 0.05/21 = 0.002), none of the SNPs retained significance although Dectin-1_rs7309123_ showed significance when the carrier status was analyzed (CC+CG *vs.* GG, *P* = 0.001; Table 3).

**Table 3:** DC-SIGN and Dectin-1 polymorphisms associated with susceptibility to Invasive Pulmonary Aspergillosis.

Gene_rs number	Genotype	IPA patients (%)	Non-IPA patients (%)	OR (95% CI)^1^	*P*-value	*P-*trend
*DC-SIGN*_rs4804800	A/A	32 (56.1)	91 (72.8)	1.00		
	A/G	21 (36.8)	31 (24.8)	2.61 (1.17–5.86)		
	G/G	4 (7.0)	3 (2.4)	3.82 (0.68–21.50)	0.031	
	A/G+G/G	25 (43.9)	34 (27.2)	2.75 (1.27–5.95)	0.009	
	per G allele			2.29 (1.21–4.35)		0.01
*DC-SIGN*_rs11465384	C/C	36 (63.2)	99 (79.2)	1.00		
	C/T	21 (36.8)	25 (20.0)	2.80 (1.27–6.15)		
	T/T	0 (0.0)	1 (0.8)	0.00 (0.00–NA)	0.029	
	C/T+T/T	21 (36.8)	26 (20.8)	2.70 (1.24–5.90)	0.012	
	per T allele			2.45 (1.16–5.17)		0.019
*DC-SIGN*_rs7248637	G/G	35 (62.5)	95 (76.6)	1.00		
	G/A	19 (33.9)	25 (20.2)	2.46 (1.09–5.58)		
	A/A	2 (3.6)	4 (3.2)	1.90 (0.27–13.41)	0.088	
	G/A+A/A	21 (37.5)	29 (23.4)	2.39 (1.09–5.22)	0.028	
	per A allele			1.94 (1.01–3.74)		0.047
*DC-SIGN*_rs7252229	G/G	30 (51.4)	79 (63.7)	1.00		
	G/C	27 (47.1)	41 (33.1)	2.50 (1.19–5.29)		
	C/C	0 (0.0)	4 (3.2)	0.00 (0.00–NA)	0.004	
	G/C+C/C	27 (47.1)	45 (36.3)	2.05 (1.00–4.22)	0.049	
	per C allele			1.49 (0.78–2.84)		0.22
*Dectin-1*_rs3901533	G/G	35 (61.4)	77 (60.7)	1.00		
	G/T	14 (24.6)	43 (33.9)	0.57 (0.24–1.36)		
	T/T	8 (14.0)	5 (5.4)	5.59 (1.37–22.77)	0.012	
	G/G+G/T	49 (86.0)	120 (96.0)	6.30 (1.56–25.37)	0.007‡	
	per T allele			1.39 (0.80–2.42)		0.25
*Dectin-1*_rs7309123	C/C	23 (40.4)	49 (39.2)	1.00		
	C/G	21 (36.8)	66 (52.8)	0.81 (0.36–1.82)		
	G/G	13 (22.8)	10 (8.0)	4.91 (1.52–15.89)	0.005	
	C/C+C/G	44 (77.2)	115 (92.0)	5.52 (1.86–16.39)	0.001‡	
	per G allele			1.75 (1.01–3.01)		0.042

1 Models adjusted for age, gender, hematological malignancy, HSCT, neutropenia (defined as absolute neutrophil count <500 cells/mm3 for a period of more than 10 days), GVHD and corticoid therapy use (>0.3 mg/Kg/day).

‡ Assuming a recessive model of inheritance. Abbreviations: OR, odds ratio; CI, confidence interval. Differences in samples numbers are due to failures in genotyping.

**Figure 1:**
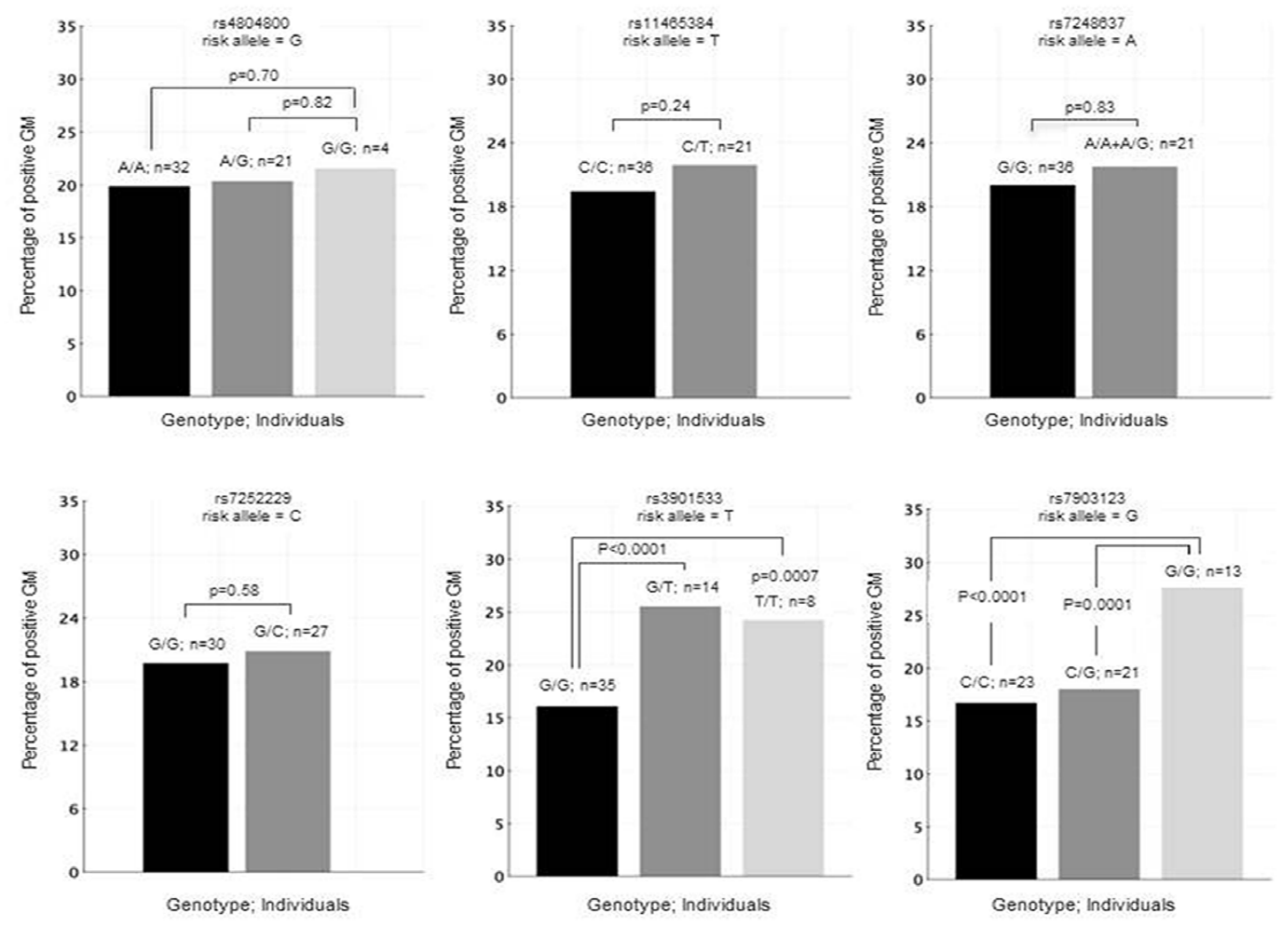
Distribution of positive galactomannan percentage by DC-SIGN and Dectin-1 genotypes.

In order to assess the degree to which the selected SNPs and the positivity of GM correlated, we also performed lineal regression analysis. In the whole population, 3784 assays were performed in duplicate and 531 were considered as positive (14.03%). We found a significantly higher percentage of positive GM among patients carrying the *Dectin-1*_rs3901533_T_ allele and among those patients bearing the *Dectin-1*_rs7309123_G/G_ genotype suggesting a role of these polymorphisms in determining a defective recognition and clearance of *Aspergillus* conidia (Figure 1). No correlation was observed for the polymorphisms within *DC-SIGN* that were associated with the infection.

We subsequently assessed whether SNPs associated with IPA infection showed capacity to change putative transcription factor binding sites using FastSNP. The predictive functional analysis suggested an intronic enhancer function for the Dectin-1_rs7309123_ SNP due to its location in transcription factor Cdxa (caudal type homeo-box transcription factor 1) binding site (GAAAGAC; score 1–2). These data suggest a central role of the rs7309123 in the susceptibility to IPA infection. None of the remaining SNPs associated with IPA infection was predicted to affect transcription factor binding sites or splicing or to introduce a damaging amino acid change.

**Figure 2:**
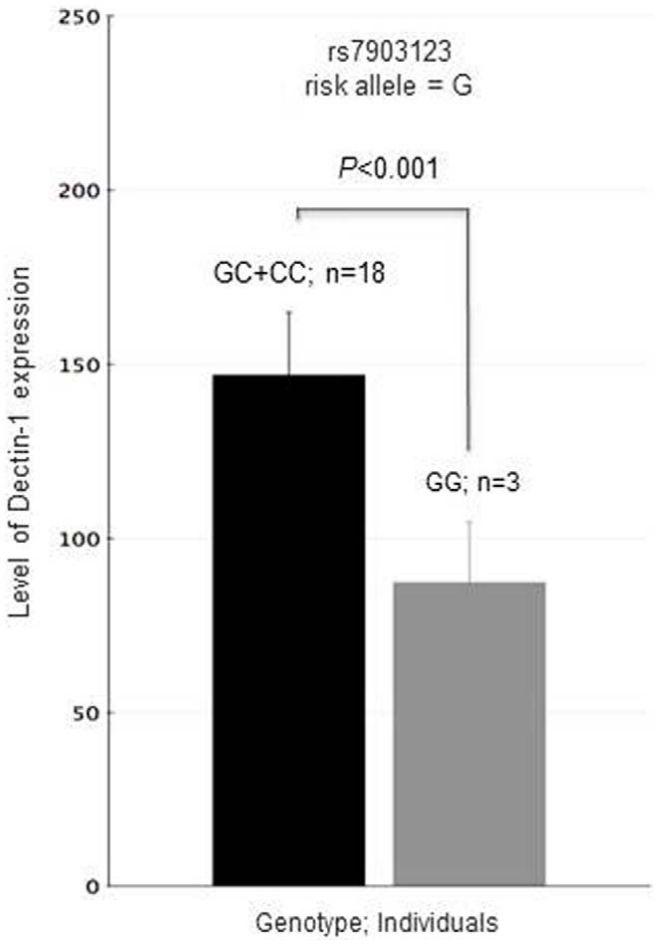
Correlation between Dectin-1_rs7903123_ genotype and expression of *Dectin-1* as measured by quantitative PCR in total RNA from healthy donors (*n* = 21). Dectin-1 mRNA levels were normalized for GAPDH mRNA levels. FirstChoice®Human Brain Reference RNA was used as Calibrator (Ambion; Catalog number AM6050).

To explore the potential consequences of Dectin-1_rs7309123_ SNP, Dectin-1 mRNA expression was measured in 21 healthy donors and was correlated with genotypes. Our results showed that subjects harbouring GG genotype showed a significantly decreased level of Dectin-1 mRNA expression compared to C-allele carriers (p<0.001; Figure 2). These results further supported our hypothesis suggesting that Dectin-1_rs7309123_G_ allele may disrupt binding sites for potential transcription factors.

**Figure 3:**
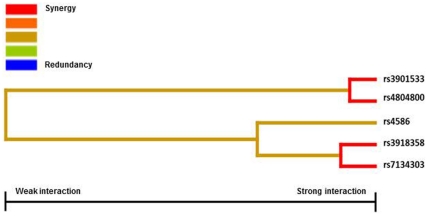
Interaction dendrogram generated by the MDR software. The interaction dendrogram was used to confirm, visualize, and interpret the interaction model. The MDR analysis was performed by using the open-source MDR software package. The colors used depict the degree of synergy, ranging from red (highest information gain) to blue (highest information redundancy). Note that the interaction between *Dectin-1* (rs3901533) and *DC-SIGN* (rs4804800) SNPs showed the highest degree of synergy (gain of information).

The MDR analysis of the SNPs tested revealed statistically significant interactions. Two SNPs (rs1024611 and rs4264222) were excluded from the MDR analysis as they were in LD with other SNPs using a cut-off value of r^2^∼0.80 in our sample set. The best interaction models selected by the TuRF filter algorithm along with its testing accuracy and cross-validation consistency are shown in Table 4. The overall best model with the highest cross-validation consistency (CVC) consisted of a model that included the *CCL2*_rs4586_, *Dectin-1*_rs3901533_, *CCR2*_rs3918358_ and *Dectin-2*_rs7134303_ SNPs. This model had a significant testing accuracy of 0.7735 (permutation p<0.001) and a cross-validation consistency of 100/100. Of note is that two SNPs showing genetic interaction in this model were not significantly associated with an increased risk of IPA infection in the univariate analysis (*CCR2*_rs3918358_ and *Dectin-2*_rs7134303_). The best two-locus model consisted of the *Dectin-1*_rs3901533_ and *DC-SIGN*_rs4804800_ SNPs, two variants that showed also association in the single-locus analysis. This model had a testing accuracy of 0.6409 and a cross-validation consistency of 76/100. This model was not any more significant after 1.000-fold permutation testing. However, the entropy based information gain calculated for this pair of SNPs indicated strong synergy, which may be interpreted as the two SNPs acting together to increase the risk of IPA infection. The best three-locus model included the *CCL2*_rs4586_, *Dectin-1*_rs3901533_ and *Dectin-2*_rs7134303_ SNPs. In this model, testing accuracy raised to 0.7085 (permutation p = 0.025) whereas the cross-validation consistency was of 68/100. Figure 3 illustrates an interaction dendogram that summarizes the estimates of interactions.

**Table 4:** Multifactor dimensionality reduction analysis summary.

	Model	TA	P-value*	CVC
1	MCP-1_rs4586	0.5881	NS	59/100
2	Dectin-1_rs3901533, DC-SIGN_rs4804800	0.6409	NS	76/100
3	MCP-1_rs4586, Dectin-1_rs3901533, Dectin-2_rs7134303	0.7085	0.025	68/100
4	MCP-1_rs4586, Dectin-1_rs3901533, CCR2_rs3918358, Dectin-2_rs7134303	0.7735	<0.001	100/100

TA, Testing accuracy; CVC, Cross-validation consistency.

* 1000-fold permutation test.

## Discussion

The marked differences in susceptibility to IPA infection among hematological patients (with or without allo-HSCT) suggest that the effective immune response against *Aspergillus* is determined by both environmental and host genetic factors [44], [45]. Studies on genetic polymorphisms in genes coding for components of the innate immunity have supported this hypothesis [19], [23]–[30], [46].

In this report, we studied the influence of the tagging and potentially functional polymorphisms of *DC-SIGN*, *Dectin-1*, *Dectin-2*, *CCL2/MCP-1* and *CCR2* in susceptibility to IPA infection in a Spanish population. Polymorphisms in these genes have been reported to influence a number of infectious diseases including HIV-1 [47]–[49], HTLV-1 [50], CMV [51], Tuberculosis [52]–[55], HCV [56], [57], HBV [58], Dengue [35] and SARS [59] among others, revealing their potential role in host defense against pathogens.

We found that polymorphisms in *Dectin-1* (rs3901533 and rs7309123) and *DC-SIGN* (rs4804800, rs11465384, rs7248637 and rs7252229) were associated with an increased risk to develop IPA infection, which points towards their critical involvement in the pathogenesis of this invasive fungal infection. The highest risk of IPA infection was found for carriers of the *Dectin-1*_rs3901533_T/T_ and *Dectin-1*_rs7309123_G/G_ genotypes and the DC-SIGN_rs4804800_G_ allele. Patients carrying these genotypes/alleles had from 2 to 6 times increased risk of IPA infection. Additionally, patients carrying the *DC-SIGN*_rs11465384_T_, *DC-SIGN*_7248637_A_ and *DC-SIGN*_rs7252229_C_ alleles showed a 2-fold increased risk in comparison with patients harboring the wild-type allele. Although it was not statistically significant, we also found that the *DC-SIGN*_rs2287886_ SNP may be associated with a reduced risk of IPA infection. Interestingly, this latter result was in agreement with our previously reported findings using the former EORTC/MSG classification criteria, 2005 [60]. The apparent effect of these SNPs on IPA susceptibility persisted even after adjustment for age, gender, hematological malignancy and several known risk factors (HSCT, neutropenia, GVHD and corticoid therapy use), indicating that *Dectin-1 and DC-SIGN* variants contribute independently to the risk of infection.

Another interesting finding of this study was the significantly greater positive GM percentage of patients carrying the *Dectin-1*_rs3901533_T_ allele than those with the wild-type allele. Additionally, patients harboring the G/G genotype for the Dectin-1_rs7903123_ SNP showed an increased percentage of positive GM compared to those carrying the C allele (C/C+C/G). No differences were found when positive GM determinations were correlated with *DC-SIGN* polymorphisms. These data along with the remarkable degree of association of *Dectin-1* and *DC-SIGN* variants with risk of IPA infection provides a compelling evidence for a critical role for these PRRs in immune response to IPA infection. In this regard, numerous studies have shown that *Dectin-1* and *DC-SIGN* are not only involved in the recognition of fungal pathogens but also in the induction of anti-fungal Th1 and Th17 immune responses [5], [11]. Mezger *et al.* also demonstrated that *Dectin-1* is involved in the induction of several pro-inflamatory cytokines, chemokines and immune receptors [18] while Werner *et al.* showed that *Dectin-1* is also regulating Th17-mediated immune response in the lungs [61]. Furthermore, Dennehy and Brown suggested a role of *Dectin-1* mediating its own signaling, as well as synergizing with TLRs to trigger NFκB-mediated immune response against fungal pathogens [62].

Although it is now well recognized that SNPs in genes modulating immune response are likely to be determinants of host susceptibility to fungal infections, so far, little is known regarding the biological significance of these variants. In order to shed light into the potential functionality of *Dectin-1* (rs3901533 and rs7309123) and *DC-SIGN* (rs4804800, rs11465384, rs7248637, rs7252229 and rs2287886) variants, we investigated whether they were involved in disruption of a binding site for critical transcription factors that might influence transcription level of these genes. Our predictive analysis showed that the carriage of the C allele for the *Dectin-1*_rs7309123_ SNP creates a putative binding site for Cdxa, a relatively unknown transcription factor, which might be involved in the control of *Dectin-1* gene expression. To assess whether the Dectin-1_rs7309123_ polymorphism might be associated with a decreased expression of Dectin-1, we correlated Dectin-1 mRNA expression levels with Dectin-1_rs7309123_ genotypes. Interestingly, we observed that individuals harbouring the GG genotype showed a relatively lower expression than those carrying the C allele (CC+GC). These results further supported our hypothesis suggesting that Dectin-1_rs7309123_ SNP may have an effect on the Dectin-1-mediated recognition of *Aspergillus* conidia and subsequent immune responses. However, this predicted change in transcription activity is only suggestive at this stage and will need further validation using *in vitro* functional assays.

Several lines of evidence point to the relevance of epistatic effects in the etiology of complex diseases but, up to now, no studies have been carried out to analyze the presence of SNP-SNP interactions in IPA infection. For this reason, we decide to assess interactions among genetic polymorphisms within *DC-SIGN*, *Dectin-1*, *Dectin-2*, *CCL2* and *CCR2*, genes and the risk of IPA infection. The MDR approach used in this study was able to determine two multilocus combinations associated with high risk to develop IPA infection. Of the interactions identified, MDR indicated that the type of interaction in the two significant models was synergistic. These results support the hypothesis that multiple SNP–SNP interactions may play a role in determining the risk of IPA infection. This hypothesis is biologically plausible since the immune system would warrant prevention of fungal infection even when some genetic variants were present.

Recent population-based studies have even led to the identification of several SNPs involved in the early recognition of *Aspergillus* and associated them with an increased risk for invasive fungal infection. It has previously been suggested that SNPs within C-type lectin genes are associated with fungal infections. Platinga *et al.* (2009) and Cunha *et al.* (2010) suggested that patients carrying the Y238X (rs16910526) polymorphism in the *Dectin-1* gene were more likely to be colonized with *Aspergillus* and *Candida* species [19], [63], compared with those harboring the wild-type allele. However, these results were not replicated in a very recent study [24]. In our study, such findings were neither evidenced even when HSCT and non-HSCT patients were analyzed separately (data not shown). This puzzling finding suggests that, although intronic polymorphisms within *Dectin-1* and *DC-SIGN* are indeed themselves a strong indication that these genes play an important role in the susceptibility to IPA infection, we cannot rule out the possibility that these SNPs are part of a bigger haplotype containing important other genetic variants in the neighboring genes. In any case, because all these population-based studies have been conducted using relatively small cohorts, additional studies in larger set of patients are needed to definitely establish the role of these variants in the susceptibility to invasive fungal infection.

In summary, this study provides evidence of association between *Dectin-1* and *DC-SIGN* polymorphisms and the risk of IPA infection. By the inclusion of functional prediction analyses, the correlation of genotypes with positive GM determinations and Dectin-1 mRNA expression levels, the study strongly supported the role of *Dectin-1* gene variants in determining susceptibility to IPA infection. Epistatic analyses also suggested the presence of a gene-gene interaction involving Dectin-1 with CCL2 and CCR2 variants to determine IPA infection. Despite all these evidences, additional studies using larger cohorts will be necessary to confirm the effect of these polymorphisms on the susceptibility to IPA infection.

## Supporting Information

Table S1 **Associations of polymorphisms involved in the phagocyte-immune related response against** ***Aspergillus*** **.** ^1^Models adjusted for age, gender, hematological malignancy, HSCT, neutropenia (defined as absolute neutrophil count <500 cells/mm3 for a period of more than 10 days), GVHD and corticoid therapy use (>0.3 mg/Kg/day). ‡ Assuming a recessive model of inheritance. Abbreviations: OR, odds ratio; CI, confidence interval. Differences in samples numbers are due to failures in genotyping.
(DOC)
