# Supplementary material for: Dectin-1 and DC-SIGN Polymorphisms Associated with Invasive Pulmonary Aspergillosis Infection
Source: PLoS One. 2012 Feb 27;7(2):e32273. doi: 10.1371/journal.pone.0032273 (PMC3288082; doi:10.1371/journal.pone.0032273)
Supplement: Table S1 — Associations of polymorphisms involved in the phagocyte-immune related response against Aspergillus . 1Models adjusted for age, gender, hematological malignancy, HSCT, neutropenia (defined as absolute neutrophil count <500 cells/mm3 for a period of more than 10 days), GVHD and corticoid therapy use (>0.3 mg/Kg/day). ‡ Assuming a recessive model of inheritance. Abbreviations: OR, odds ratio; CI, confidence interval. Differences in samples numbers are due to failures in genotyping. (DOC) [file pone.0032273.s001.doc]

**Table S1.** Associations of polymorphisms involved in the phagocyte-immune related response against *Aspergillus*.

| **Gene_rs number** | **Genotype** | **IPA patients (%)** | **Non-IPA patients (%)** | **OR (95 CI)^1^** | ***p*-value** | ***p* trend** |
| --- | --- | --- | --- | --- | --- | --- |
|  |  |  |  |  |  |  |
| DC-SIGN_rs2287886 |  |  |  |  |  |  |
|  | G/G | 30 (52.6) | 50 (40.3) | 1.00 |  |  |
|  | A/G | 23 (40.4) | 62 (50.0) | 0.60 (0.29-1.27) |  |  |
|  | A/A | 4 (7.0) | 12 (9.7) | 0.36 (0.10-1.34) | 0.190 |  |
|  | A/G+A/A | 27 (47.4) | 74 (59.7) | 0.55 (0.27-1.13) | 0.100 |  |
|  | per A allele |  |  | 0.60 (0.34-1.05) |  | 0.069 |
|  |  |  |  |  |  |  |
| DC-SIGN_rs4804803 |  |  |  |  |  |  |
|  | A/A | 28 (49.1) | 70 (56.0) | 1.00 |  |  |
|  | A/G | 26 (45.6) | 44 (35.2) | 2.01 (0.95-4.27) |  |  |
|  | G/G | 3 (5.3) | 11 (8.8) | 0.56 (0.12-2.52) | 0.096 |  |
|  | A/G+G/G | 29 (50.9) | 55 (44.0) | 1.62 (0.80-3.29) | 0.180 |  |
|  | per G allele |  |  | 1.17 (0.68-2.02) |  | 0.570 |
|  |  |  |  |  |  |  |
| DC-SIGN_rs4804800 |  |  |  |  |  |  |
|  | A/A | 32 (56.1) | 91 (72.8) | 1.00 |  |  |
|  | A/G | 21 (36.8) | 31 (24.8) | 2.61 (1.17-5.86) |  |  |
|  | G/G | 4 (7.0) | 3 (2.4) | 3.82 (0.68-21.50) | 0.031 |  |
|  | A/G+G/G | 25 (43.9) | 34 (27.2) | 2.75 (1.27-5.95) | 0.009 |  |
|  | per G allele |  |  | 2.29 (1.21-4.35) |  | 0.009 |
|  |  |  |  |  |  |  |
| DC-SIGN_rs8112310 |  |  |  |  |  |  |
|  | T/T | 35 (61.4) | 77 (62.1) | 1.00 |  |  |
|  | A/T | 19 (33.3) | 43 (34.7) | 1.01 (0.48-2.12) |  |  |
|  | A/A | 3 (5.3) | 4 (3.2) | 2.11 (0.35-12.69) | 0.720 |  |
|  | A/T+A/A | 22 (38.6) | 47 (37.9) | 1.10 (0.54-2.23) | 0.800 |  |
|  | per A allele |  |  | 1.16 (0.63-2.14) |  | 0.620 |
|  |  |  |  |  |  |  |
| DC-SIGN_rs10410342 |  |  |  |  |  |  |
|  | G/G | 53 (93.0) | 109 (87.2) | 1.00 |  |  |
|  | C/G | 4 (7.0) | 13 (10.4) | 1.08 (0.28-4.14) |  |  |
|  | C/C | 0 (0.0) | 2 (2.4) | 0.00 (0.00-NA) | 0.100 |  |
|  | C/G+C/C | 4 (7.0) | 16 (12.8) | 0.61 (0.17-2.14) | 0.430 |  |
|  | per C allele |  |  | 0.52 (0.19-1.46) |  | 0.190 |
|  |  |  |  |  |  |  |
| DC-SIGN_rs11465384 |  |  |  |  |  |  |
|  | C/C | 36 (63.2) | 99 (79.2) | 1.00 |  |  |
|  | C/T | 21 (36.8) | 25 (20.0) | 2.80 (1.27-6.15) |  |  |
|  | T/T | 0 (0.0) | 1 (0.8) | 0.00 (0.00-NA) | 0.029 |  |
|  | C/T+T/T | 21 (36.8) | 26 (20.8) | 2.70 (1.24-5.90) | 0.012 |  |
|  | per T allele |  |  | 2.45 (1.16-5.17) |  | 0.019 |
|  |  |  |  |  |  |  |
| DC-SIGN_rs11465413 |  |  |  |  |  |  |
|  | T/T | 42 (73.7) | 103 (82.4) | 1.00 |  |  |
|  | A/T | 14 (24.6) | 22 (17.6) | 1.20 (0.50-2.86) |  |  |
|  | A/A | 1 (1.8) | 0 (0.0) | 0.00 (0.00-NA) | 0.410 |  |
|  | A/T+A/A | 15 (26.3) | 22 (17.6) | 1.28 (0.55-3.02) | 0.570 |  |
|  | per A allele |  |  | 1.37 (0.61-3.10) |  | 0.450 |
|  |  |  |  |  |  |  |
| DC-SIGN_rs7252229 |  |  |  |  |  |  |
|  | G/G | 30 (51.4) | 79 (63.7) | 1.00 |  |  |
|  | G/C | 27 (47.1) | 41 (33.1) | 2.50 (1.19-5.29) |  |  |
|  | C/C | 0 (0.0) | 4 (3.2) | 0.00 (0.00-NA) | 0.004 |  |
|  | G/C+C/C | 27 (47.1) | 45 (36.3) | 2.05 (1.00-4.22) | 0.049 |  |
|  | per C allele |  |  | 1.49 (0.78-2.84) |  | 0.220 |
|  |  |  |  |  |  |  |
| DC-SIGN_rs7248637 |  |  |  |  |  |  |
|  | G/G | 35 (62.5) | 95 (76.6) | 1.00 |  |  |
|  | G/A | 19 (33.9) | 25 (20.2) | 2.46 (1.09-5.58) |  |  |
|  | A/A | 2 (3.6) | 4 (3.2) | 1.90 (0.27-13.41) | 0.088 |  |
|  | G/A+A/A | 21 (37.5) | 29 (23.4) | 2.39 (1.09-5.22) | 0.028 |  |
|  | per A allele |  |  | 1.94 (1.01-3.74) |  | 0.047 |
|  |  |  |  |  |  |  |
| DC-SIGN_rs11465421 |  |  |  |  |  |  |
|  | A/A | 14 (24.6) | 30 (24.0) | 1.00 |  |  |
|  | A/C | 30 (52.6) | 68 (54.4) | 1.11 (0.48-2.57) |  |  |
|  | C/C | 13 (22.8) | 27 (21.6) | 1.23 (0.44-3.46) | 0.930 |  |
|  | A/C+C/C | 43 (75.4) | 95 (76.0) | 1.14 (0.51-2.55) | 0.740 |  |
|  | per C allele |  |  | 1.11 (0.66-1.86) |  | 0.690 |
|  |  |  |  |  |  |  |
| Dectin-1_rs16910526 |  |  |  |  |  |  |
|  | A/A | 46 (83.6) | 102 (82.3) | 1.00 |  |  |
|  | A/C | 9 (16.4) | 22 (17.7) | 1.14 (0.42-3.15) | 0.790 |  |
|  |  |  |  |  |  | - |
|  |  |  |  |  |  |  |
| Dectin-1_rs11053599 |  |  |  |  |  |  |
|  | C/C | 41 (77.4) | 84 (70.6) | 1.00 |  |  |
|  | A/C | 11 (20.8) | 33 (27.7) | 0.90 (0.37-2.15) |  |  |
|  | A/A | 1 (1.9) | 2 (1.7) | 0.85 (0.07-10.68) | 0.960 |  |
|  | A/C+A/A | 12 (22.6) | 35 (29.4) | 0.89 (0.38-2.07) | 0.790 |  |
|  | per A allele |  |  | 0.90 (0.43-1.90) |  | 0.790 |
|  |  |  |  |  |  |  |
| Dectin-1_rs7309123 |  |  |  |  |  |  |
|  | C/C | 23 (40.4) | 49 (39.2) | 1.00 |  |  |
|  | C/G | 21 (36.8) | 66 (52.8) | 0.81 (0.36-1.82) |  |  |
|  | G/G | 13 (22.8) | 10 (8.0) | 4.91 (1.52-15.89) | 0.005 |  |
|  | C/C+C/G | 44 (77.2) | 115 (92.0) | 5.52 (1.86-16.39) | 0.001 ‡ |  |
|  | per G allele |  |  | 1.75 (1.01-3.01) |  | 0.042 |
|  |  |  |  |  |  |  |
| Dectin-1_rs3901533 |  |  |  |  |  |  |
|  | G/G | 35 (61.4) | 77 (60.7) | 1.00 |  |  |
|  | G/T | 14 (24.6) | 43 (33.9) | 0.57 (0.24-1.36) |  |  |
|  | T/T | 8 (14.0) | 5 (5.4) | 5.59 (1.37-22.77) | 0.012 |  |
|  | G/G+G/T | 49 (86.0) | 120 (96.0) | 6.30 (1.56-25.37) | 0.007 ‡ |  |
|  | per T allele |  |  | 1.39 (0.80-2.42) |  | 0.250 |
|  |  |  |  |  |  |  |
| Dectin-1_rs4763446 |  |  |  |  |  |  |
|  | T/T | 41 (73.2%) | 97 (78.2%) | 1 |  |  |
|  | C/T | 13 (23.2%) | 25 (20.2%) | 1.07 (0.45-2.51) |  |  |
|  | C/C | 2 (3.6%) | 2 (1.6%) | 2.91 (0.29-29.23) | 0.660 |  |
|  | C/T+C/C | 15 (26.8%) | 27 (21.8%) | 1.18 (0.52-2.67) | 0.700 |  |
|  | per C allele |  |  | 1.25 (0.61-2.55) |  | 0.540 |
|  |  |  |  |  |  |  |
| Dectin-1_rs16910631 |  |  |  |  |  |  |
|  | C/C | 46 (80.7%) | 103 (82.4%) | 1 |  |  |
|  | C/T | 11 (19.3%) | 22 (17.6%) | 1.13 (0.44-2.90) | 0.810 |  |
|  |  |  |  |  |  | - |
|  |  |  |  |  |  |  |
| Dectin-1_rs7311598 |  |  |  |  |  |  |
|  | A/A | 48 (84.2%) | 96 (76.8%) | 1 |  |  |
|  | A/G | 8 (14%) | 26 (20.8%) | 0.70 (0.27-1.84) |  |  |
|  | G/G | 1 (1.8%) | 3 (2.4%) | 0.72 (0.05-9.48) | 0.760 |  |
|  | A/G+G/G | 9 (15.8%) | 29 (23.2%) | 0.71 (0.28-1.78) | 0.450 |  |
|  | per G allele |  |  | 0.75 (0.33-1.68) |  | 0.480 |
|  |  |  |  |  |  |  |
| Dectin-2_rs7134303 |  |  |  |  |  |  |
|  | A/A | 38 (66.7%) | 67 (54%) | 1 |  |  |
|  | A/G | 17 (29.8%) | 49 (39.5%) | 0.61 (0.28-1.31) |  |  |
|  | G/G | 2 (3.5%) | 8 (6.5%) | 0.28 (0.05-1.67) | 0.190 |  |
|  | A/G+G/G | 19 (33.3%) | 57 (46%) | 0.55 (0.26-1.15) | 0.110 |  |
|  | per G allele |  |  | 0.57 (0.31-1.07) |  | 0.072 |
|  |  |  |  |  |  |  |
| Dectin-2_rs4264222 |  |  |  |  |  |  |
|  | C/C | 37 (64.9%) | 62 (49.6%) | 1 |  |  |
|  | C/T | 18 (31.6%) | 55 (44%) | 0.58 (0.27-1.22) |  |  |
|  | T/T | 2 (3.5%) | 8 (6.4%) | 0.28 (0.05-1.67) | 0.160 |  |
|  | C/T+T/T | 20 (35.1%) | 63 (50.4%) | 0.53 (0.26-1.09) | 0.086 |  |
|  | per T allele |  |  | 0.56 (0.30-1.03) |  | 0.056 |
|  |  |  |  |  |  |  |
| Dectin-2_rs4459385 |  |  |  |  |  |  |
|  | C/C | 28 (50.9%) | 77 (62.6%) | 1 |  |  |
|  | C/T | 22 (40%) | 38 (30.9%) | 1.46 (0.69-3.10) |  |  |
|  | T/T | 5 (9.1%) | 8 (6.5%) | 2.14 (0.54-8.50) | 0.420 |  |
|  | C/T+T/T | 27 (49.1%) | 46 (37.4%) | 1.55 (0.76-3.17) | 0.230 |  |
|  | per T allele |  |  | 1.46 (0.83-2.57) |  | 0.190 |
|  |  |  |  |  |  |  |
| CCL2_rs4586 |  |  |  |  |  |  |
|  | T/T | 29 (50.9%) | 48 (38.4%) | 1 |  |  |
|  | C/T | 18 (31.6%) | 61 (48.8%) | 0.58 (0.26-1.26) |  |  |
|  | C/C | 10 (17.5%) | 16 (12.8%) | 1.24 (0.43-3.57) | 0.26 |  |
|  | C/T+C/C | 28 (49.1%) | 77 (61.6%) | 0.72 (0.35-1.45) | 0.35 |  |
|  | per C allele |  |  | 0.95 (0.57-1.56) |  | 0.82 |
|  |  |  |  |  |  |  |
| CCL2_rs1024610 |  |  |  |  |  |  |
|  | A/A | 31 (54.4%) | 70 (56%) | 1 |  |  |
|  | T/A | 22 (38.6%) | 46 (36.8%) | 1.24 (0.59-2.62) |  |  |
|  | T/T | 4 (7%) | 9 (7.2%) | 1.16 (0.30-4.51) | 0.85 |  |
|  | T/A+T/T | 26 (45.6%) | 55 (44%) | 1.23 (0.60-2.49) | 0.57 |  |
|  | per T allele |  |  | 1.15 (0.66-1.99) |  | 0.63 |
|  |  |  |  |  |  |  |
| CCL2_rs1024611 |  |  |  |  |  |  |
|  | A/A | 32 (56.1%) | 63 (50.4%) | 1 |  |  |
|  | G/A | 24 (42.1%) | 53 (42.4%) | 1.17 (0.57-2.41) |  |  |
|  | G/G | 1 (1.8%) | 9 (7.2%) | 0.24 (0.03-2.20) | 0.29 |  |
|  | G/A+G/G | 25 (43.9%) | 62 (49.6%) | 1.01 (0.50-2.01) | 0.98 |  |
|  | per G allele |  |  | 0.87 (0.48-1.56) |  | 0.63 |
|  |  |  |  |  |  |  |
| CCL2_rs13900 |  |  |  |  |  |  |
|  | C/C | 32 (57.1%) | 61 (48.8%) | 1 |  |  |
|  | C/T | 23 (41.1%) | 56 (44.8%) | 1.02 (0.50-2.10) |  |  |
|  | T/T | 1 (1.8%) | 8 (6.4%) | 0.23 (0.02-2.11) | 0.32 |  |
|  | C/T+T/T | 24 (42.9%) | 64 (51.2%) | 0.88 (0.44-1.78) | 0.73 |  |
|  | per T allele |  |  | 0.79 (0.43-1.43) |  | 0.43 |
|  |  |  |  |  |  |  |
| CCR2_rs3918358 |  |  |  |  |  |  |
|  | A/A | 26 (45.6%) | 67 (53.6%) | 1 |  |  |
|  | A/C | 28 (49.1%) | 46 (36.8%) | 1.78 (0.83-3.80) |  |  |
|  | C/C | 3 (5.3%) | 12 (9.6%) | 0.62 (0.12-3.22) | 0.22 |  |
|  | A/C+C/C | 58 (46.4%) | 58 (46.4%) | 1.54 (0.74-3.17) | 0.24 |  |
|  | per C allele |  |  | 1.19 (0.67-2.11) |  | 0.56 |
|  |  |  |  |  |  |  |
| CCR2_rs743660 |  |  |  |  |  |  |
|  | G/G | 30 (53.6%) | 73 (59.4%) | 1 |  |  |
|  | A/G | 24 (42.9%) | 40 (32.5%) | 1.55 (0.73-3.28) |  |  |
|  | A/A | 2 (3.6%) | 10 (8.1%) | 0.55 (0.09-3.51) | 0.360 |  |
|  | A/G+A/A | 26 (46.4%) | 50 (40.6%) | 1.38 (0.67-2.85) | 0.390 |  |
|  | per A allele |  |  | 1.13 (0.62-2.06) |  | 0.690 |
|  |  |  |  |  |  |  |
| CCR2_rs1799864 |  |  |  |  |  |  |
|  | G/G | 50 (87.7%) | 103 (82.4%) | 1 |  |  |
|  | A/G | 7 (12.3%) | 21 (16.8%) | 0.73 (0.27-1.99) |  |  |
|  | A/A | 0 (0%) | 1 (0.8%) | 0.00 (0.00-NA) | 0.40 |  |
|  | A/G+A/A | 7 (12.3%) | 22 (17.6%) | 0.67 (0.25-1.81) | 0.42 |  |
|  | per A allele |  |  | 0.63 (0.25-1.62) |  | 0.33 |
|  |  |  |  |  |  |  |

^1^Models adjusted for age, gender, hematological malignancy, HSCT, neutropenia (defined as absolute neutrophil count <500 cells/mm3 for a period of more than 10 days), GVHD and corticoid therapy use (>0.3mg/Kg/day). ‡ Assuming a recessive model of inheritance. Abbreviations: OR, odds ratio; CI, confidence interval. Differences in samples numbers are due to failures in genotyping. Results in bold show p<0.05.
